# Supplementary material for: STRategies to manage Emergency ambulance Telephone Callers with sustained High needs: an Evaluation using linked Data (STRETCHED) – a study protocol
Source: BMJ Open. 2022 Mar 29;12(3):e053123. doi: 10.1136/bmjopen-2021-053123 (PMC8966558; doi:10.1136/bmjopen-2021-053123)
Supplement: Author's manuscript [file bmjopen-2021-053123.draft_revisions.pdf]

BMJ Open

BMJ Open is committed to open peer review. As part of this commitment we make the peer review history of every article we publish publicly available.

When an article is published we post the peer reviewers' comments and the authors' responses online. We also post the versions of the paper that were used during peer review. These are the versions that the peer review comments apply to.

The versions of the paper that follow are the versions that were submitted during the peer review process. They are not the versions of record or the final published versions. They should not be cited or distributed as the published version of this manuscript.

BMJ Open is an open access journal and the full, final, typeset and author-corrected version of record of the manuscript is available on our site with no access controls, subscription charges or pay-per-view fees (http://bmjopen.bmj.com).

If you have any questions on BMJ Open's open peer review process please email info.bmjopen@bmj.com

BMJ Open

STRategies to manage Emergency ambulance Telephone Callers with sustained High needs – an Evaluation using linked Data (STRETCHED): study protocol

Journal:	*BMJ Open*
Manuscript ID	bmjopen-2021-053123
Article Type:	Protocol
Date Submitted by the Author:	28-Jul-2021
Complete List of Authors:	Aslam, Rabeeah; Swansea University, PRIME Centre Wales<br>Snooks, Helen; Swansea University, Medicine<br>Porter, Alison; University of Swansea, College of Medicine<br>Khanom, Ashrafunnesa; Swansea University, School of Medicine<br>Cole, Robert; West Midlands Ambulance Service NHS Trust<br>Edwards, Adrian; Cardiff University,<br>Edwards, Bethan; Swansea University<br>Evans, Bridie; Swansea University, Medicine<br>Foster, Theresa; East of England Ambulance Service NHS Trust<br>Fothergill, Rachael; London Ambulance Service NHS Trust,<br>Gripper, Penny; Swansea University, PRIME Centre Wales<br>John, Ann; Swansea University, Medical sChool<br>Petterson, Robin; Welsh Ambulance Services NHS Trust<br>Rosser, Andy; West Midlands Ambulance Service NHS Foundation Trust<br>Tee, Anna; Swansea University, PRIME Centre Wales<br>Sewell, Bernadette; Swansea University, College of Human and Health Sciences<br>Hughes, Heather; Swansea University, PRIME Centre Wales<br>Phillips, Ceri; Swansea University, Human and Health Sciences<br>Rees, Nigel; Welsh Ambulance Service NHS Trust, Pre Hospital Emergency Research Unit<br>Scott, Jason; Northumbria University, Faculty of health and life sciences<br>Watkins, Alan; Swansea University, College of Medicine
Keywords:	ACCIDENT & EMERGENCY MEDICINE, HEALTH SERVICES ADMINISTRATION & MANAGEMENT, MENTAL HEALTH, Chronic airways disease < THORACIC MEDICINE, Epilepsy < NEUROLOGY, DIABETES & ENDOCRINOLOGY

SCHOLARONE™
Manuscripts

Title

STRategies to manage Emergency ambulance Telephone Callers with sustained High needs – an Evaluation using linked Data (STRETCHED): study protocol

Corresponding Author

Dr. Rabeea'h W Aslam

Swansea University, PRIME Centre Wales
Swansea University Medical School
Institute of Life Sciences 2, Floor 2
West Glamorgan
Swansea, UK SA2 8PP
07900857741

r.w.aslam@swansea.ac.uk

Co-authors

Dr. Rabeea'h Waseem Aslam<br>Swansea University Medical School, Singleton Park, Swansea, SA1 8PP	r.w.aslam@swansea.ac.uk
Professor Helen A Snooks<br>Swansea University Medical School, Singleton Park, Swansea, SA1 8PP	h.a.snooks@swansea.ac.uk
Associate Professor Alison Porter<br>Swansea University Medical School, Singleton Park, Swansea, SA1 8PP	a.m.porter@swansea.ac.uk
Ashrafunnesa Khanom<br>Swansea University Medical School, Singleton Park, Swansea, SA1 8PP	a.khanom@swansea.ac.uk
Robert Cole<br>West Midlands Ambulance Service Trust University NHS Foundation Trust , Waterfront Business Park, Brierley Hill, West Midlands DY5 1LX	robert.cole@wmas.nhs.uk
Professor Adrian Edwards<br>Division of Population Medicine School of Medicine, Cardiff University, Cardiff, CF10 3AT	EdwardsAG@cardiff.ac.uk
Bethan Mair Edwards<br>Swansea University Medical School, Singleton Park, Swansea, SA1 8PP	BethanMairEdwards@hotmail.co.uk
Dr Bridie A Evans<br>Swansea University Medical School, Singleton Park, Swansea, SA1 8PP	B.A.Evans@Swansea.ac.uk
Theresa Foster<br>East of England Ambulance Service, Milford Service Area, Barton Mills, Suffolk, IP28 6AE	theresa.foster@eastamb.nhs.uk
Rachael T Fothergill<br>London Ambulance Service, 220 Waterloo Rd, Lambeth, London SE1 8SD	Rachael.fothergill1@nhs.net
Gripper, Penny<br>Swansea University Medical School, Singleton Park, Swansea, SA1 8PP	cpennygripper@googlemail.com
Professor Ann John<br>Swansea University Medical School, Singleton Park, Swansea, SA1 8PP	A.John@Swansea.ac.uk
Robin Petterson<br>Welsh Ambulance Services NHS Trust Headquarters, St Asaph Business Park, Denbighshire LL17 0LJ	Robin.Petterson@wales.nhs.uk
Andy Rosser<br>West Midlands Ambulance Service Trust University NHS Foundation Trust, Waterfront Business Park, Brierley Hill, West Midlands DY5 1LX	andy.rosser@wmas.nhs.uk
Anna Tee<br>Swansea University Medical School, Singleton Park, Swansea, SA1 8PP	Annastee71@gmail.com

For peer review only - http://bmjopen.bmj.com/site/about/guidelines.xhtml

Dr Bernadette Sewell<br>Swansea Centre for health Economics, College of Human and Health Sciences, Swansea University, Singleton Park, Swansea, SA1 8PP	b.diethart@swansea.ac.uk
Heather Hughes<br>Swansea University Medical School, Singleton Park, Swansea, SA1 8PP	Hev.hughes@ntlworld
Ceri J Phillips<br>Swansea University Medical School, Singleton Park, Swansea, SA1 8PP	C.J.Phillips@Swansea.ac.uk
Nigel Rees<br>Welsh Ambulance Service NHS Trust, Health & Care Research Wales	Nigel Rees<br>Nigel.Rees5@wales.nhs.uk
Dr Jason Scott<br>Northumbria University, Sutherland Building, Newcastle-upon-Tyne NE1 8ST	jason.scott@northumbria.ac.uk
Professor Alan Watkins<br>Swansea University Medical School, Singleton Park, Swansea, SA1 8PP	a.watkins@swansea.ac.uk

Word count: 3,448

ABSTRACT

Introduction

The NHS is under sustained pressure, particularly in unscheduled emergency and urgent care, with 999 calls increasing by 6% every year. All UK ambulance services have identified a clinical and operational concern with high users of the 999 service and have set up 'Frequent Callers' services, ranging from within-service management to cross-sectoral multi-disciplinary case management approaches. These callers are known to have a range of health and care needs including mental health crises. There is little evidence about how to address the needs of this patient group effectively and to appropriately reduce frequent contacts in this setting.

Research Aim: To evaluate effectiveness, safety, and efficiency of case management approaches to the care of people who frequently call the emergency ambulance service; and gain an understanding of barriers and facilitators to implementation.

Research Objectives:

1. Develop an understanding of predicted mechanisms of change to underpin evaluation
2. Describe epidemiology of sustained high users of 999 services
3. Evaluate case management approaches to the care of people who call the 999 ambulance service frequently in terms of:
    - further emergency contacts (999, Emergency Department, emergency admissions to hospital)
    - effects on other services
    - adverse events (deaths; injuries; serious medical emergencies; police arrests)
    - costs of intervention and care

For peer review only - http://bmjopen.bmj.com/site/about/guidelines.xhtml

For peer review only

- patient experience of care

4. Identify challenges and opportunities associated with using case management models, including features associated with success, and develop theories about how case management works in this population

Methods

We will conduct a multisite mixed methods evaluation of case management for people who use ambulance services frequently by using anonymised linked routine data outcomes in a 'natural experiment' cohort design, in four regional ambulance services with one case management intervention site and one control site within each service. We will conduct interviews and focus groups with service users, commissioners, and emergency and non-acute care providers regarding acceptability, successes and challenges.

Ethics

The study received approval from the Health Research Authority (Confidentiality Advisory Group reference: 19/CAG/0195; Research Ethics Committee reference: 19/WA/0216) in the United Kingdom

Strengths and limitations of this study

- Use of privacy-protected routinely collected anonymised linked data will provide an efficient, inclusive and holistic picture of service use
- Impact likely to be high as study is closely aligned to current health and social care policy agenda
- Collaborative patient focus with public involvement throughout study
- Quasi-experimental study design not as strong as prospective randomised trial
- Quantitative health outcomes are based on routine data with no self-reported quality of life outcomes

For peer review only - http://bmjopen.bmj.com/site/about/guidelines.xhtml

INTRODUCTION

Background

Pressures on emergency ambulance services are growing at an unsustainable rate in Europe and North America [1,2,3].The escalation of emergency calls to the ambulance services presents as a major operational challenge [4,5]. The volume of emergency calls to ambulance services in England doubled from 4.72 million in 2001/02, to 9 million in 2014/15 [1], and national performance targets were not met for 32 consecutive [6].

Emergency ambulance services provide care for those with urgent and life-threatening health conditions. Although most people make few calls to the emergency ambulance service, a small minority of patients make more intensive use [7]. Unresolved health or social care needs within this group put additional pressure on already stretched emergency ambulance services, which were originally designed to respond to patients with time-critical needs for clinical intervention, rather than to manage non-acute care needs.

Definitions have varied [8, 9] but UK ambulance services agree that people who make five or more calls per month or 12 calls over a three months period should be classified as 'frequent callers'. In London, of a total of 1.7 million calls made during 2014–15, 1,622 people met 'frequent caller' criteria and generated 49,534 ambulance attendances, at an estimated cost of £4.4 million to the ambulance service [10].

Research shows that people who make high use of emergency services also experience higher mortality rates than those with lower use of services [11]. People who call the emergency ambulance service frequently are often vulnerable, [7, 12, 13, 4] more likely to be from low socio-economic group live alone, experience mental health challenges including self-harming behaviour, live with chronic conditions, and have increased chances of falling [14,15]. Repeated access of services and calling may be because problems are unresolved or patients are not aware of an alternative pathway to access appropriate care [7,16].

Taking lessons from initiatives in primary care to incentivise the prioritisation of care of frail older patients who are at risk of emergency admission to hospital [12], commissioners in the UK now require ambulance services to have management strategies in place for people who call frequently [13, 17]. In some areas case management approaches are in place, with comprehensive care plans developed for patients who call the emergency ambulance service frequently, through multi-disciplinary, cross-sector management groups. Key decision-makers from commissioning, acute, primary, secondary and charitable health and social care

For peer review only - http://bmjopen.bmj.com/site/about/guidelines.xhtml

providers meet with ambulance service staff to share, stratify risk and manage patients in regular Multi-Disciplinary Team (MDT) meetings.

The introduction of case management has the potential to support a change to safe and equitable out-of-hospital care for this patient group, and to avoid patients being shifted to another part of the emergency care system, wider NHS or social care without their care needs being addressed, but research is needed to evaluate this new approach.

Research Aim

To evaluate effectiveness, efficiency, safety and patient experience of case management approaches to the care of people who call the emergency ambulance service frequently; and gain an understanding of barriers and facilitators to implementation.

Research Objectives

1. Develop an understanding of predicted mechanisms of change to underpin the evaluation
2. Describe epidemiology of people who use 999 frequently
3. Evaluate case management approaches to the care of people who call the 999-ambulance service frequently in terms of:
    - further emergency contacts (emergency ambulance calls, Emergency Department attendance, emergency admissions to hospital)
    - effects on other health and social care services
    - adverse events (deaths; injuries; serious medical emergencies; police arrests)
    - costs of intervention and subsequent use of health and social care
    - patient experience of intervention and subsequent use of health and social care
4. Identify challenges and opportunities associated with using case management models, including features associated with success, and develop theories about how case management works in this population.

METHODS AND ANALYSIS

Study design

STRategies to manage Emergency ambulance Telephone Callers with sustained High needs – an Evaluation using linked Data (STRETCHED) is a mixed methods 'natural experiment' evaluation. We will use anonymised linked routine outcomes and qualitative data in four UK ambulance services with one intervention and one control site in each service.

For peer review only - http://bmjopen.bmj.com/site/about/guidelines.xhtml

Work package 1: Logic model

We will develop a logic model in consultation with stakeholders during a face-to-face workshop to underpin the evaluation and inform data collection, in particular, finalisation of outcome selection. The logic model will include definition of key components of case management in this setting; predicted mechanisms of change; and possible outcomes, positive or otherwise. Stakeholders to be invited to this workshop include clinical and managerial partners working within the ambulance service, Emergency Departments, primary care and high intensity user services from across the UK.

Figure 1 shows a draft logic model, developed from the research literature and specialist knowledge of research team members, to be used as a basis for discussion and development during WP1.

Work package 2: Evaluation

We will undertake an evaluation using a natural experiment cohort design in four ambulance services, using quantitative anonymised linked routine data to describe epidemiology and assess effects of the intervention on processes, outcomes, safety and costs of intervention and subsequent health and social care up to six months later, with adjustment for covariates including prior service use. We will also collect qualitative data from focus groups and interviews in each intervention site about the views and experiences of stakeholders (commissioners, emergency and non-acute health and social care providers) regarding acceptability, successes and challenges of case management approaches for this group of patients; and in-depth interviews with a range of service users within the target population

Work package 3: Synthesis of quantitative and qualitative findings

We will formally synthesise quantitative and qualitative findings from WP 2, informed by the logic model developed in WP1 and in consultation with stakeholders included in the research team

For peer review only

Setting

The study will be undertaken in four ambulance services, identified through a survey of practice across the UK, where both case management and traditional 'within-service' models are in place in different areas within the service. Emergency ambulance services in the UK provide emergency are to '999' callers which is free at the point of use. Following mergers in the 1990s, services cover large areas, each serving a population of between 3 and 9 million people; most cover urban and rural locations, and provide a range of emergency responses including telephone advice, attendance of an emergency vehicle for face to face assessment by a paramedic or Emergency Medical Technician, and conveyance to hospital for further care when judged clinically appropriate.

Characteristics of control and intervention site care in the four ambulance service sites included in STRETCHED are summarised in Table 1.

Table 1: Sites within participating services and key features of control (usual) and intervention (case management) site care

	Control site: Usual care	Intervention site: New model of care
Care models: generic description	Aim: to reduce or stop calls made by people making high use of the 999 service<br><br>Within-service management<br>• Letter sent to GP and patient<br>• Callers flagged, care management plan developed for use in ambulance call centre to triage patient when call comes in<br>• Contact may be made with other services to intervene<br>• Police action may be requested	Aim: to address and meet complex patient needs, thereby reducing emergency contacts by people making high use of the 999<br><br>Multi-Disciplinary Team cross-sector case management<br>Usual (within-service) care plus<br>• Dedicated frequent caller nurse/prehospital care practitioner funded by CCG/HB<br>• Multidisciplinary team meetings attended by ambulance service; partnership approach with other agencies including district nursing, social workers, police, out of hours providers, mental health professionals, ED, voluntary sector, NHS 111, GPs and Occupational Therapy to develop and share joint care plans to address patient need

For peer review only - http://bmjopen.bmj.com/site/about/guidelines.xhtml

Outcomes

1. Further emergency contacts:
    - Emergency ambulance calls
    - Emergency Department attendance
    - Emergency admissions to hospital
    - Declassification/reclassification as 'frequent caller'
2. Effects on other health and social care services
3. Adverse events, as available
    - Deaths
    - Injuries
    - Serious medical emergencies
    - Police arrests
4. Costs of intervention and subsequent use of health and social care
5. Patient experience of intervention

Data collection

We will use a parallel cohort 'natural experiment' study design to determine effects on processes and outcomes of care, using anonymised linked data from NHS Digital and the NHS Wales Informatics Service (NWIS). The variation in exposure and outcomes will allow us to carry out analysis to link effects to intervention i.e. causes [1]. We will include callers meeting the criteria for classification as 'frequent caller' by the ambulance service during 2018. We expect to recruit 300 high users per service (n=1200), allowing us to detect a change in rate of further emergency events/death of +/- 20% with 90% power and 95% confidence (see figure 2). We will finalise data items following completion of the logic model but expect to include (up to six months): further calls to emergency ambulance service, ED attendances, emergency admissions, and deaths; declassification or reclassification as 'frequent caller'; costs; and details of demographics, casemix and patterns of calls, e.g. 'out-of-hours' (evenings/nights/weekends/holidays). Historical data about prior service use will allow us to adjust analyses for differences between cohorts, strengthening this study design.

We will collect qualitative data at the intervention site within each participating ambulance service to explore perceptions of how the intervention works, what creates its effect (if any), why it might function differently in different settings and for different groups of people, and any challenges to implementation and delivery of the intervention. Subject to any restrictions associated with COVID-19, we will conduct four focus groups (supplemented where necessary by telephone or online interviews) with stakeholders – including front line staff (paramedics, call handlers), partner health and social care providers, commissioners and managers (n=36). Focus group topic guides will cover case management delivery processes, barriers and facilitators to changed working, perceived impact for patients, issues around diversity and terminology, strengths and weaknesses of the approach and wider organisational

For peer review only - http://bmjopen.bmj.com/site/about/guidelines.xhtml

impact across health economies, such as information sharing, communication and continuity of care.

To ensure we gain a strong picture of patient experience and circumstances we will conduct largely unstructured interviews with people in intervention areas of each service who are referred for case management (n=32). Research paramedics based at each site will undertake interviews as far as possible. We will use this method to provide an opportunity for patients to provide their own narrative about their circumstances, experiences and views regarding their needs, service use and care received before and after the case management intervention, and the intervention itself, including terminology used. We will work with participating services to identify and invite callers to this key patient-focused element of the study. We will sample purposively in order to include a wide range of views and experiences, and select individuals with differing demographic characteristics, length of time they have required care and management approach provided for them to include typical and atypical patient stories. We will try to include patients from Black, Asian and ethnic minority groups.

With respondents' consent, we will audio-record and transcribe all individual and group interviews.

Data analysis

Quantitative analysis

Ambulance services will provide NHS Digital and NHS Wales Informatics Service (NWIS) with information on recruited patients, to allow matching to anonymised data from multiple sources. We will create a single integrated study database for analysis within the Secure Anonymised Information Linkage (SAIL) Gateway (19).

We will summarise patient recruitment via a CONSORT flowchart [20], and will provide descriptive data summaries for patients, by service and by site, for demographics, call patterns and volume. Numbers of events will be dichotomised where contextually useful (for instance, to declassify or reclassify callers), and converted to rates when appropriate.

Our primary analyses will be by treatment allocated, so that patients meeting the criteria for 'frequent caller' status in an intervention (case management) site will be included in analysis in that arm of the study whether or not they were offered or received any intervention. The outcome measure time point will be six months from a patient's first appearance on a monthly update to the 'frequent callers' list. This will apply to patients at control sites as well as intervention sites. This time point is appropriate to allow us to detect any effect, as case management is a targeted, time limited intervention designed to work within six months.

We will use multilevel generalised mixed linear models to obtain adjusted comparison of outcomes in patients at intervention sites versus those in patients receiving usual care at control sites. Using linked routine data will allow us to gather retrospective

For peer review only - http://bmjopen.bmj.com/site/about/guidelines.xhtml

data on service use for callers included in each cohort, strengthening comparisons by enabling adjustment for historical and contemporaneous differences in service use, casemix, and demographics. Reflecting an initial focus on comparing (any) case management approach with the service list approach, coding for sites will initially assume that outcomes under usual care are similar across ambulance services, as are outcomes at intervention sites. However, we will use background information on the service interventions, the descriptive summaries and formal testing, based on flexible and nested site coding, to test and modify this assumption, as appropriate – for instance, allowing intervention outcomes to vary systematically from service to service. The precise form of models will reflect the nature of the variable under consideration (logistic models for binary variables; negative binomial models for count variables; linear models for raw and transformed measurement outcomes, including rates); the multilevel element of the model will reflect the geographical clustering of sites within services. The adjusted comparisons will incorporate information on covariates and factors based on demographic and case-mix data. We will formalise these analyses in a Statistical Analysis Plan, using the relevant Swansea Trials Unit Standard Operating Procedure, which will detail conventions on model fitting (including inclusion and exclusion rules for covariates and factors), management of missing data, and the reporting of outcomes.

Health Economic evaluation

Using a cost-benefit framework, we will compare the implementation cost of case management approaches to the potential benefits of "avoiding expenditure" in subsequent health and social care resource utilisation from a public sector perspective. The overall resource implications for case management approaches for frequent callers will include intervention implementation costs, costs of 999 calls and the costs arising from utilisation of other health and care services in the 6-month follow-up period.

The costs of case management interventions will be derived through discussion with relevant staff and observation of operational processes and procedures, taking into account staff time and other resources required to deliver the intervention Each resource element will be costed using appropriate unit costs derived from published sources, and/or consultation with relevant finance staff.

The costs associated with a sample of the 999 calls in each study area will be determined by identifying staff inputs, along with materials, equipment, therapies and other relevant resources used during the response to the call and the processes involved in its management and completion. A cost profile for 999 calls will be developed for each geographical area, and summarised as cost per 999 call.

The utilisation of other health and care services by the study cohorts in the control and intervention sites will be captured by routine data sources (i.e. NHS Digital and the SAIL databank), and costed using appropriate published unit costs. The inclusion of mental health and social care resources will be subject to routine data availability and quality validation.

For peer review only - http://bmjopen.bmj.com/site/about/guidelines.xhtml

In order to assess the rate of return on investment, we will describe changes in service utilisation over time, using changes in volume of calls and their occurrence. Our cost-benefit analysis will include net present value and internal rate of return estimates to assess the relative value for money of case management approaches in managing frequent callers of ambulance 999 services and whether the cost of case management services are shared across organisations. We will undertake a series of sensitivity analyses to estimate the effect of parameter variation on baseline findings and to determine the extent to which case management of these callers is an efficient use of public funds.

Qualitative analysis:

Analysis of focus group and interview transcripts will be carried out by members of the research team with the two Public and Patient Involvement (PPI) representatives, alongside input from the research paramedics. We will remove all identifiable data from interview transcripts before analysis. We will use a data-driven thematic approach to analysis which generates themes from the implicit and explicit ideas within participants' accounts [21]. We will follow the six stages of analysis described by Braun and Clarke [22]: data familiarisation, generating initial coding, searching for themes, reviewing themes, defining and naming themes, producing a report. During the analysis process, we will check emerging coding and themes with the wider research team and members of the patient panel. We will assess data saturation [23,24,25] during analysis to see whether new themes are emerging by reviewing the codebook (the working document that records updates in changes to codes). We will also assess whether we have an adequate range of participants reflected in our purposive sample. Analysis of the large volume of data (transcripts, field and observation notes) generated by the interviews will be supported by use of NVivo, computer assisted qualitative data analysis software.

Synthesis

Synthesis and reporting of quantitative and qualitative findings will be informed by the STRETCHED logic model. Quantitative data will be used to draw conclusions about comparative costs and effects; qualitative data will help us to understand and interpret these results, and to generate theories about how the new models of care are working. We will bring together key themes from across all the work packages on the effectiveness, attitudes, barriers and facilitators to case management. We will interpret overall effectiveness and cost-effectiveness results in the light of analysis from the qualitative data about which components of case management e.g. care plan, timing of interventions, shared decision-making are perceived to work well and for whom. We will use the logic model to inform the synthesis of results which will be considered and interpreted at a joint meeting of the Research Management and Patient Advisory Groups.

ETHICS

We have received approval from the Health Research Authority (19/WA/0216) and NHS R&D permissions at all participating organisations.

For peer review only - http://bmjopen.bmj.com/site/about/guidelines.xhtml

Patients will not be approached for consent to participate in the main effectiveness study (WP2) as identifying information will be held within ambulance services only and not shared with the research team. With information governance permissions in place, retrospective routine data will be linked anonymously by NHS Digital and NWIS using a split file approach, for analysis within the SAIL Gateway [26].

A small sample of current patients who are calling frequently will be invited by the participating ambulance services to give consent and participate in one to one interviews. We recognise that these patients may be vulnerable and will take care to ensure that interviews are carried out sensitively, in a place of the respondent's choice or by telephone/online e.g. Zoom and by appropriately trained researchers at each site.

PATIENT AND PUBLIC INVOLVEMENT

We have worked extensively with public contributors to develop a comprehensive approach to active involvement of patients and the public at all study stages [25]. We are proposing a layered approach so that people can be involved at strategic and local levels in line with their interest, experience and health. Our aim is to enable active and meaningful involvement throughout to enhance research quality, rigour and ethical standards, in line with the Swansea Trials Unit SOP on service user involvement [25] and best practice [27]. Our public co-applicants (PG, BE) have been actively involved in study design and shaping the proposal. They have been involved in proposal design to undertake interviews with patients, challenging biases and assumptions in the team and helping choose of proper language to describe people who phone 999. They are members of the Research Management Group and equal partners in decisions about study implementation and dissemination.

We will also convene a Patient Advisory Panel of six individuals recruited through community groups, support agencies and third sector networks. The Panel will reflect the range of people who make frequent calls to 999 including older people, those with chronic illness and people from lower socio-economic levels. We will seek to ensure that public contributors within the study match as closely as possible the diverse population of people making frequent calls. This Panel will be a less formal route for people to contribute to specific tasks such as advising on patient facing materials, sense-checking patient results and devising dissemination materials. We will hold face-to-face or virtual meetings and visit people in their homes if that is their preference, in order to ensure their involvement (25, 27, 28).

We will recruit a further two public members to the independent Research Advisory Group to bring patient perspectives to oversight and scrutiny decisions. We will offer honoraria for all involvement and reimburse incurred expenses.
We will provide training for all public contributors, proposing an induction at start of involvement and training in specific skills such as Good Clinical Practice, analysis, meeting skills and dissemination skills, as contributors require.

For peer review only - http://bmjopen.bmj.com/site/about/guidelines.xhtml

DISCUSSION

Evaluation of interventions that have already been implemented is challenging. It is impossible to carry out randomised controlled trials in this circumstance, frequently encountered in the real world of health services research [31]. In this circumstance alternative evaluation designs need to be used, inevitably sacrificing the ability to definitively ascribe changes observed to the intervention being evaluated – as there is always the possibility of confounding by other systematic differences between sites or patients. In the STRETCHED study we have planned a quasi-experimental study in which we will compare differences between control sites (usual care) combined and intervention (case management) sites combined. Using linked anonymised outcomes we will be able to adjust for pervious patterns of service utilisation, thus strengthening our comparisons.

It is important to provide evidence about what works and how for this patient group, in order to inform the development of care. We aim, through this study, to gain robust evidence about costs, effects, safety and stakeholder views, including patients so that the best care can be provided to improve processes and outcomes for people who make high use of the emergency ambulance service, as well as to reduce pressure on services that provide emergency care.

Competing Interests: There are no competing interests for any authors

Authors' contributions: All authors contributed to the development of the protocol

Funding statement: This project is funded by the National Institute for Health Research (NIHR) Health Services & Delivery Research (HS&DR) Programme (reference 18/03/02). The views expressed are those of the author(s) and not necessarily those of the NIHR or the Department of Health and Social Care

For peer review only - http://bmjopen.bmj.com/site/about/guidelines.xhtml

REFERENCES

1. The Health and Social Care Information Centre (HSCIC). Ambulance services, England 2014-15. Available from: https://digital.nhs.uk/data-and-information/publications/statistical/ambulance-services/ambulance-services-england-2014-15 (accessed 10 Aug 2020).
2. Emily Andrew, Ziad Nehme, Peter Cameron & Karen Smith (2020) Drivers of Increasing Emergency Ambulance Demand, Prehospital Emergency Care, 24:3, 385, DOI: 10.1080/10903127.2019.1635670
3. Burt CW, McCaig LF, Valverde RH. Analysis of ambulance transports and diversions among US emergency departments. Ann Emerg Med 2006; 47: 317–326.
4. Association of ambulance chief executives. Annual Report 2014-15. Available from: http://aace.org.uk/wp-content/uploads/2015/10/AACE-ANNUAL-REPORT-2014-2015-FINAL-W.pdf.(accessed 10 Aug 2020).
5. Keogh B and Urgent and Emergency Care Review Team. Transforming urgent and emergency care services in England Urgent and Emergency Care Review End of Phase 1 Report NHS England. Leeds; 2013. Available from: https://www.nhs.uk/NHSEngland/keogh-review/Documents/UECR.Ph1Report.FV.pdf. (accessed 10 Aug 2020).
6. Ambulance Quality Indicators; NHS England. November 2016. Available from: https://www.england.nhs.uk/statistics/statistical-work-areas/ambulance-quality-indicators/ (accessed 10 Aug 2020).
7. Edwards M, Bassett G, Sinden L et al. Frequent callers to the ambulance service: patient profiling and impact of case management on patient utilisation of the ambulance service. Emergency Medicine Journal 2014;32:392-396. doi:10.1136/emermed-2013-203496
8. Smith D. Frequent caller: identification and management policy. North west ambulance service NHS trust; 2015. Available from: https://www.nwas.nhs.uk/media/387174/frequent_callers_policy_final_v2_0_ds.pdf. (accessed 10 Aug 2020).
9. Scott, J., Strickland, A. P., Warner, K., & Dawson, P. (2014). Frequent callers to and users of emergency medical systems: a systematic review. Emergency medicine journal, 31(8), 684-691.
10. The Health and Social Care Information Centre (HSCIC). Ambulance services, England 2014-15. Available from: https://digital.nhs.uk/data-and-information/publications/statistical/ambulance-services/ambulance-services-england-2014-15 (accessed 10 Aug 2020).
11. Moe J, Kirkland S, Ospina M et al. Mortality, admission rates and outpatient use among frequent users of emergency departments: a systematic review. Emergency Medicine Journal 2015;33:230-236. doi:10.1136/emermed-2014-204496
12. Althaus F, Paroz S, Hugli O et al. Effectiveness of Interventions Targeting Frequent Users of Emergency Departments: A Systematic Review. Annals of Emergency Medicine 2011;58:41-52.e42. doi:10.1016/j.annemergmed.2011.03.007

For peer review only - http://bmjopen.bmj.com/site/about/guidelines.xhtml

13. Gravelle H, Dusheiko M, Sheaff R et al. Impact of case management (Evercare) on frail elderly patients: controlled before and after analysis of quantitative outcome data. BMJ 2006;334:31. doi:10.1136/bmj.39020.413310.55
14. Agarwal, G., Lee, J., McLeod, B., Mahmuda, S., Howard, M., Cockrell, K., & Angeles, R. (2019). Social factors in frequent callers: a description of isolation, poverty and quality of life in those calling emergency medical services frequently. BMC public health, 19(1), 684.
15. Mahmuda, S., Wade-Vallance, A., Stosic, A., Guenter, D., Howard, M., Agarwal, G., ... & Angeles, R. (2020). Understanding Why Frequent Users of EMS Call 9-1-1: A Grounded Theory Study. Health Promotion Practice, 21(3), 440-447.
16. Scott J, Strickland A, Warner K et al. Describing and predicting frequent callers to an ambulance service: analysis of 1 year call data. Emergency Medicine Journal 2013;31:408-414. doi:10.1136/emermed-2012-202146
17. Moe J, Kirkland S, Ospina M et al. Mortality, admission rates and outpatient use among frequent users of emergency departments: a systematic review. Emergency Medicine Journal 2015;33:230-236. doi:10.1136/emermed-2014-204496
18. Wassmer R, Winward L, Derlet R. Does Counseling Reduce Frequent Emergency Department Use?. 2008. Available from: https://papers.ssrn.com/abstract=1093870 (accessed 10 Aug 2020).
19. Jones, K. H., Ford, D. V., Thompson, S. and Lyons, R. (2020) "A Profile of the SAIL Databank on the UK Secure Research Platform", International Journal of Population Data Science, 4(2). doi: 10.23889/ijpds.v4i2.1134.
20. Schulz K, Altman D, Moher D. CONSORT 2010 Statement: updated guidelines for reporting parallel group randomised trials. BMC Medicine 2010;8. doi:10.1186/1741-7015-8-18
21. Guest G, MacQueen K, Namey E. Applied thematic analysis. Los Angeles: : Sage Publications 2012.
22. Braun V, Clarke V. Using thematic analysis in psychology. Qualitative Research in Psychology 2006;3:77-101. doi:10.1191/1478088706qp063oa.
23. Marshall C, Rossman G. Designing qualitative research. Los Angeles: : Sage 2016.
24. Ziebland S, McPherson A. Making sense of qualitative data analysis: an introduction with illustrations from DIPEx (personal experiences of health and illness). Medical Education 2006;40:405-414. doi:10.1111/j.1365-2929.2006.02467.x
25. Miles MB, Huberman AM. Qualitative Data Analysis. An Expanded Sourcebook. Second edition. Thousand Oaks, CA: Sage 1994
26. Lyons R, Jones K, John G et al. The SAIL databank: linking multiple health and social care datasets. BMC Medical Informatics and Decision Making 2009;9. doi:10.1186/1472-6947-9-3
27. Staniszewska S, Brett J, Simera I, Seers K, Mockford C, Goodland S et al. GRIPP2 reporting checklists: tools to improve reporting of patient and public involvement in research BMJ 2017; 358 :j3453
28. Evans B, Bedson E, Bell P et al. Involving service users in trials: developing a standard operating procedure. Trials 2013;14:219. doi:10.1186/1745-6215-14-219

For peer review only - http://bmjopen.bmj.com/site/about/guidelines.xhtml

29. Crowe, S., Adebajo, A., Esmael, H. et al. 'All hands-on deck', working together to develop UK standards for public involvement in research. Res Involv Engagem 6, 53 (2020). https://doi.org/10.1186/s40900-020-00229-y
30. INVOLVE – What is public involvement in research? http://www.invo.org.uk/find-out-more/what-is-public-involvement-in-research-2/. (accessed 10 Aug 2020)
31. Deaton, A., & Cartwright, N. (2018). Understanding and misunderstanding randomized controlled trials. Social science & medicine (1982), 210, 2–21. https://doi.org/10.1016/j.socscimed.2017.12.005

Figure 1 Emergent logic model

Figure 2 'Natural Experiment' Flow Diagram

For peer review only

For peer review only - http://bmjopen.bmj.com/site/about/guidelines.xhtml

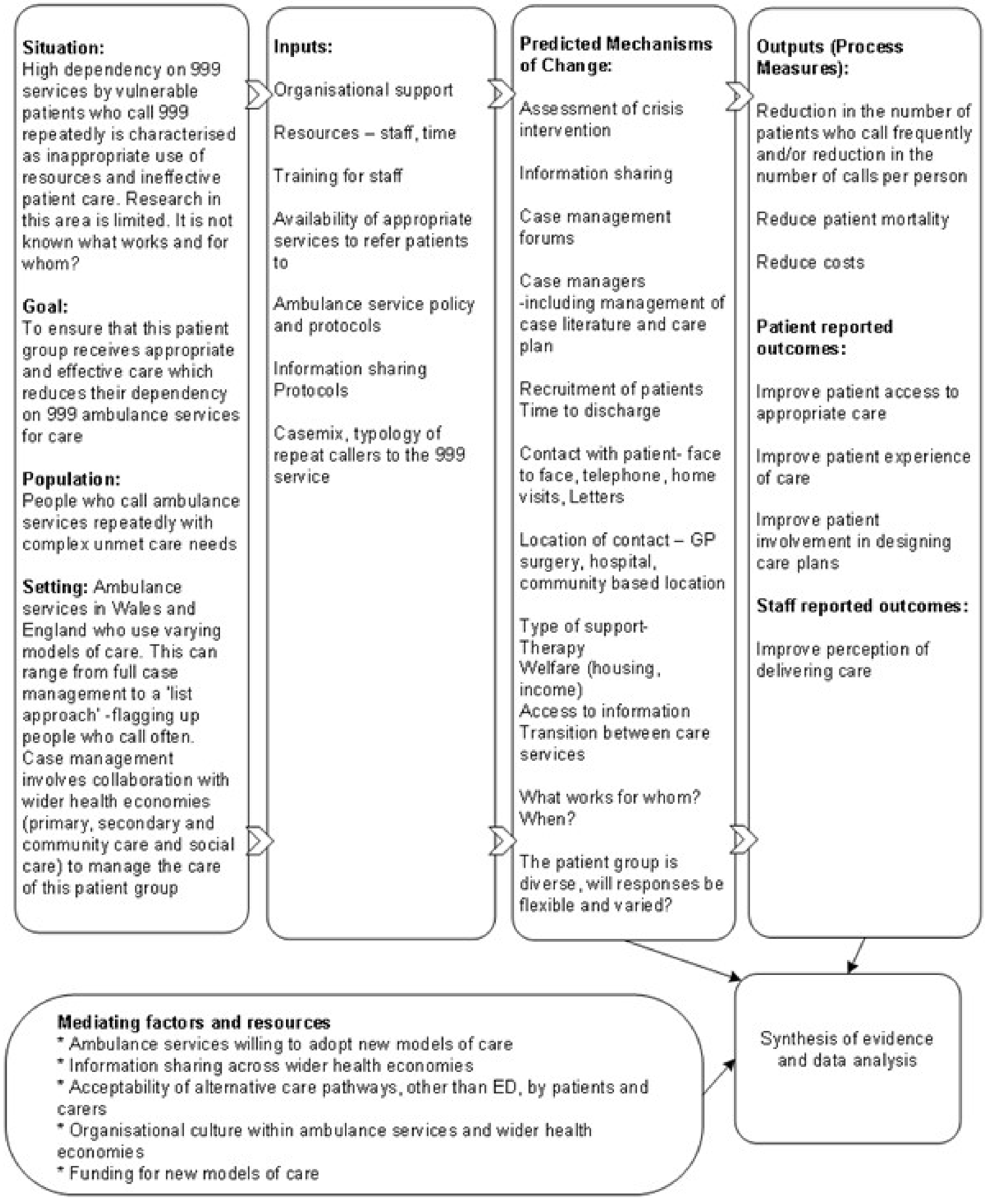

Figure 1 Emergent logic model

159x193mm (96 x 96 DPI)

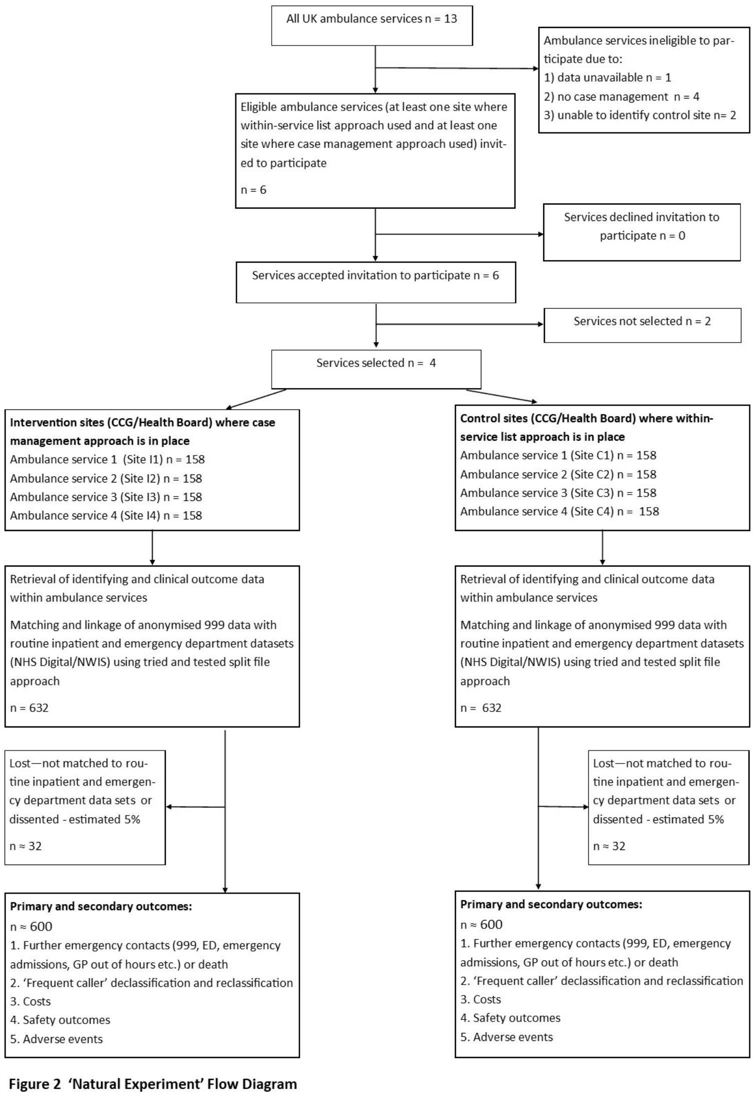

Figure 2 'Natural Experiment' Flow Diagram

209x297mm (150 x 150 DPI)

CONSORT 2010 checklist of information to include when reporting a randomised trial*

Section/Topic	Item No	Checklist item	Reported on page No
**Title and abstract**			
	1a	Identification as a randomised trial in the title	1
	1b	Structured summary of trial design, methods, results, and conclusions (for specific guidance see CONSORT for abstracts)	2
**Introduction**			
Background and objectives	2a	Scientific background and explanation of rationale	3
	2b	Specific objectives or hypotheses	4
**Methods**			
Trial design	3a	Description of trial design (such as parallel, factorial) including allocation ratio	3
	3b	Important changes to methods after trial commencement (such as eligibility criteria), with reasons	N/A
Participants	4a	Eligibility criteria for participants	7
	4b	Settings and locations where the data were collected	7,8
Interventions	5	The interventions for each group with sufficient details to allow replication, including how and when they were actually administered	7
Outcomes	6a	Completely defined pre-specified primary and secondary outcome measures, including how and when they were assessed	8
	6b	Any changes to trial outcomes after the trial commenced, with reasons	N/A
Sample size	7a	How sample size was determined	9
	7b	When applicable, explanation of any interim analyses and stopping guidelines	9, 10
Randomisation:			N/A
Sequence generation	8a	Method used to generate the random allocation sequence	7
	8b	Type of randomisation; details of any restriction (such as blocking and block size)	7
Allocation concealment mechanism	9	Mechanism used to implement the random allocation sequence (such as sequentially numbered containers), describing any steps taken to conceal the sequence until interventions were assigned	N/A
Implementation	10	Who generated the random allocation sequence, who enrolled participants, and who assigned participants to interventions	N/A
Blinding	11a	If done, who was blinded after assignment to interventions (for example, participants, care providers, those	

For peer review only

For peer review only - http://bmjopen.bmj.com/site/about/guidelines.xhtml

		assessing outcomes) and how	
	11b	If relevant, description of the similarity of interventions	7
Statistical methods	12a	Statistical methods used to compare groups for primary and secondary outcomes	9,10
	12b	Methods for additional analyses, such as subgroup analyses and adjusted analyses	10
**Results**			
Participant flow (a diagram is strongly recommended)	13a	For each group, the numbers of participants who were randomly assigned, received intended treatment, and were analysed for the primary outcome	9,10
	13b	For each group, losses and exclusions after randomisation, together with reasons	Attached figure 2
Recruitment	14a	Dates defining the periods of recruitment and follow-up	9
	14b	Why the trial ended or was stopped	N/A
Baseline data	15	A table showing baseline demographic and clinical characteristics for each group	N/A
Numbers analysed	16	For each group, number of participants (denominator) included in each analysis and whether the analysis was by original assigned groups	N/A
Outcomes and estimation	17a	For each primary and secondary outcome, results for each group, and the estimated effect size and its precision (such as 95% confidence interval)	10
	17b	For binary outcomes, presentation of both absolute and relative effect sizes is recommended	N/A
Ancillary analyses	18	Results of any other analyses performed, including subgroup analyses and adjusted analyses, distinguishing pre-specified from exploratory	N/A
Harms	19	All important harms or unintended effects in each group (for specific guidance see CONSORT for harms)	N/A
**Discussion**			
Limitations	20	Trial limitations, addressing sources of potential bias, imprecision, and, if relevant, multiplicity of analyses	9,10
Generalisability	21	Generalisability (external validity, applicability) of the trial findings	9,10
Interpretation	22	Interpretation consistent with results, balancing benefits and harms, and considering other relevant evidence	9,10
**Other information**			
Registration	23	Registration number and name of trial registry	N/A
Protocol	24	Where the full trial protocol can be accessed, if available	N/A
Funding	25	Sources of funding and other support (such as supply of drugs), role of funders	16

*We strongly recommend reading this statement in conjunction with the CONSORT 2010 Explanation and Elaboration for important clarifications on all the items. If relevant, we also recommend reading CONSORT extensions for cluster randomised trials, non-inferiority and equivalence trials, non-pharmacological treatments, herbal interventions, and pragmatic trials. Additional extensions are forthcoming: for those and for up to date references relevant to this checklist, see www.consort-statement.org.

For peer review only - http://bmjopen.bmj.com/site/about/guidelines.xhtml

BMJ Open

STRategies to manage Emergency ambulance Telephone Callers with sustained High needs – an Evaluation using linked Data (STRETCHED): study protocol

Journal:	*BMJ Open*
Manuscript ID	bmjopen-2021-053123.R1
Article Type:	Protocol
Date Submitted by the Author:	24-Jan-2022
Complete List of Authors:	Aslam, Rabeeah; Swansea University, PRIME Centre Wales<br>Snooks, Helen; Swansea University, Medicine<br>Porter, Alison; University of Swansea, College of Medicine<br>Khanom, Ashrafunnesa; Swansea University, School of Medicine<br>Cole, Robert; West Midlands Ambulance Service NHS Trust<br>Edwards, Adrian; Cardiff University,<br>Edwards, Bethan; Swansea University<br>Evans, Bridie; Swansea University, Medicine<br>Foster, Theresa; East of England Ambulance Service NHS Trust<br>Fothergill, Rachael; London Ambulance Service NHS Trust,<br>Gripper, Penny; Swansea University, PRIME Centre Wales<br>John, Ann; Swansea University, Medical sChool<br>Petterson, Robin; Welsh Ambulance Services NHS Trust<br>Rosser, Andy; West Midlands Ambulance Service NHS Foundation Trust<br>Tee, Anna; Swansea University, PRIME Centre Wales<br>Sewell, Bernadette; Swansea University, College of Human and Health Sciences<br>Hughes, Heather; Swansea University, PRIME Centre Wales<br>Phillips, Ceri; Swansea University, Human and Health Sciences<br>Rees, Nigel; Welsh Ambulance Service NHS Trust, Pre Hospital Emergency Research Unit<br>Scott, Jason; Northumbria University, Faculty of health and life sciences<br>Watkins, Alan; Swansea University, College of Medicine
**Primary Subject Heading**:	Emergency medicine
Secondary Subject Heading:	Health services research, Mental health
Keywords:	ACCIDENT & EMERGENCY MEDICINE, HEALTH SERVICES ADMINISTRATION & MANAGEMENT, MENTAL HEALTH, Chronic airways disease < THORACIC MEDICINE, Epilepsy < NEUROLOGY, DIABETES & ENDOCRINOLOGY

Title

STRategies to manage Emergency ambulance Telephone Callers with sustained High needs – an Evaluation using linked Data (STRETCHED): study protocol

Corresponding Author

Dr Rabeea'h W Aslam

Swansea University, PRIME Centre Wales
Swansea University Medical School
Institute of Life Sciences 2, Floor 2
West Glamorgan
Swansea, UK SA2 8PP
07900857741

r.w.aslam@swansea.ac.uk

Co-authors

Rabeea'h Waseem Aslam<br>Swansea University Medical School, Singleton Park, Swansea, SA1 8PP	r.w.aslam@swansea.ac.uk
Professor Helen A Snooks<br>Swansea University Medical School, Singleton Park, Swansea, SA1 8PP	h.a.snooks@swansea.ac.uk
Associate Professor Alison Porter<br>Swansea University Medical School, Singleton Park, Swansea, SA1 8PP	a.m.porter@swansea.ac.uk
Ashrafunnesa Khanom<br>Swansea University Medical School, Singleton Park, Swansea, SA1 8PP	a.khanom@swansea.ac.uk
Robert Cole<br>West Midlands Ambulance Service Trust University NHS Foundation Trust, Waterfront Business Park, Brierley Hill, West Midlands DY5 1LX	robert.cole@wmas.nhs.uk
Professor Adrian Edwards<br>Division of Population Medicine School of Medicine, Cardiff University, Cardiff, CF10 3AT	EdwardsAG@cardiff.ac.uk
Bethan Mair Edwards<br>Swansea University Medical School, Singleton Park, Swansea, SA1 8PP	BethanMairEdwards@hotmail.co.uk
Dr Bridie A Evans<br>Swansea University Medical School, Singleton Park, Swansea, SA1 8PP	B.A.Evans@Swansea.ac.uk
Theresa Foster<br>East of England Ambulance Service, Milford Service Area, Barton Mills, Suffolk, IP28 6AE	theresa.foster@eastamb.nhs.uk
Rachael T Fothergill<br>London Ambulance Service, 220 Waterloo Rd, Lambeth, London SE1 8SD	Rachael.fothergill1@nhs.net
Gripper, Penny<br>Swansea University Medical School, Singleton Park, Swansea, SA1 8PP	cpennygripper@googlemail.com
Professor Ann John<br>Swansea University Medical School, Singleton Park, Swansea, SA1 8PP	A.John@Swansea.ac.uk
Robin Petterson<br>Welsh Ambulance Services NHS Trust Headquarters, St Asaph Business Park, Denbighshire LL17 0LJ	Robin.Petterson@wales.nhs.uk
Andy Rosser<br>West Midlands Ambulance Service Trust University NHS Foundation Trust, Waterfront Business Park, Brierley Hill, West Midlands DY5 1LX	andy.rosser@wmas.nhs.uk
Anna Tee<br>Swansea University Medical School, Singleton Park, Swansea, SA1 8PP	Annastee71@gmail.com

For peer review only

Dr Bernadette Sewell<br>Swansea Centre for health Economics, College of Human and Health Sciences, Swansea University, Singleton Park, Swansea, SA1 8PP	b.diethart@swansea.ac.uk
Heather Hughes<br>Swansea University Medical School, Singleton Park, Swansea, SA1 8PP	Hev.hughes@ntlworld
Ceri J Phillips<br>Swansea University Medical School, Singleton Park, Swansea, SA1 8PP	C.J.Phillips@Swansea.ac.uk
Nigel Rees<br>Welsh Ambulance Service NHS Trust, Health & Care Research Wales	Nigel Rees Nigel.Rees5@wales.nhs.uk
Dr Jason Scott<br>Northumbria University, Sutherland Building, Newcastle-upon-Tyne NE1 8ST	jason.scott@northumbria.ac.uk
Professor Alan Watkins<br>Swansea University Medical School, Singleton Park, Swansea, SA1 8PP	a.watkins@swansea.ac.uk

Word count: 3,448

For peer review only

ABSTRACT

Introduction

UK ambulance services have identified a concern with high users of the 999 service and have set up 'Frequent Callers' services, ranging from within-service management to cross-sectoral multi-disciplinary case management approaches. There is little evidence about how to address the needs of this patient

Aim

To evaluate effectiveness, safety, and efficiency of case management approaches to the care of people who frequently call the emergency ambulance service; and gain an understanding of barriers and facilitators to implementation. Objectives:

1. Develop an understanding of predicted mechanisms of change to underpin evaluation
2. Describe epidemiology of sustained high users of 999 services
3. Evaluate case management approaches to the care of people who call the 999-ambulance service frequently in terms of:
    a. further emergency contacts (999, Emergency Department, emergency admissions to hospital)
    b. effects on other services
    c. adverse events (deaths; injuries; serious medical emergencies; police arrests)
    d. costs of intervention and care
    e. patient experience of care
4. Identify challenges and opportunities associated with using case management models, including features associated with success, and develop theories about how case management works in this population

Methods and analysis

We will conduct a multisite mixed-methods evaluation of case management for people who use ambulance services frequently by using anonymised linked routine data outcomes in a 'natural experiment' cohort design, in four regional ambulance services. We will conduct interviews and focus groups with service users, commissioners, and emergency and non-acute care providers. The planned start and end date of the study is 01.04.2019 and 01.09.2022 respectively

Ethics and dissemination

The study received approval from the UK Health Research Authority (Confidentiality Advisory Group reference: 19/CAG/0195; Research Ethics Committee reference: 19/WA/0216)

For peer review only - http://bmjopen.bmj.com/site/about/guidelines.xhtml

We will collate feedback from our Lived Experience Advisory Panel, the Frequent Caller National Network, Research Management Group for targeted dissemination activities.

Article Summary

Strengths and limitations of this study

1. Use of privacy-protected routinely collected anonymised linked data will provide an efficient, inclusive, and holistic picture of service use
2. Impact likely to be high as study is closely aligned to current health and social care policy agenda
3. Collaborative patient-focus with public involvement throughout study
4. Quasi-experimental study design not as strong as prospective randomised trial
5. Quantitative health outcomes are based on routine data with no self-reported quality of life outcomes

INTRODUCTION

Background

Pressures on emergency ambulance services are growing at an unsustainable rate in Europe and North America [1,2,3]. The escalation of emergency calls to the ambulance services presents as a major operational challenge [4,5]. The volume of emergency calls to ambulance services in England doubled from 4.72 million in 2001/02, to 9 million in 2014/15 [1], and national performance targets were not met for 32 consecutively [6].

Emergency ambulance services provide care for those with urgent and life-threatening health conditions. Although most people make few calls to the emergency ambulance service, a small minority of patients make more intensive use [7]. Unresolved health or social care needs within this group put additional pressure on already stretched emergency ambulance services, which were originally designed to respond to patients with time-critical needs for clinical intervention, rather than to manage non-acute care needs.

Definitions have varied [8, 9] but UK ambulance services agree that people who make five or more calls per month or 12 calls over a three month period should be classified as ‘frequent callers. In London, of a total of 1.7 million calls made during 2014–15, 1,622 people met ‘frequent caller’ criteria and generated 49,534 ambulance attendances, at an estimated cost of £4.4 million to the ambulance service [10].

For peer review only - http://bmjopen.bmj.com/site/about/guidelines.xhtml

Research shows that people who make high use of emergency services also experience higher mortality rates than those with lower use of services [11]. People who call the emergency ambulance service frequently are often vulnerable, [7, 12, 13, 4] more likely to be from low socio-economic group live alone, experience mental health challenges including self-harming behaviour, live with chronic conditions, and have increased chances of falling [14,15]. Repeated access of services and calling may be because problems are unresolved or patients are not aware of an alternative pathway to access appropriate care [7,16].

Taking lessons from initiatives in primary care to incentivise the prioritisation of care of frail older patients who are at risk of emergency admission to hospital [12], commissioners in the UK now require ambulance services to have management strategies in place for people who call frequently [13, 17]. In some areas case management approaches are in place, with comprehensive care plans developed for patients who call the emergency ambulance service frequently, through multi-disciplinary, cross-sector management groups. Key decision-makers from commissioning, acute, primary, secondary, and charitable health and social care providers meet with ambulance service staff to share, stratify risk, and manage patients in regular Multi-Disciplinary Team (MDT) meetings[18].

The introduction of case management has the potential to support a change to safe and equitable out-of-hospital care for this patient group, and to avoid patients being shifted to another part of the emergency care system, wider NHS or social care without their care needs being addressed, but research is needed to evaluate this new approach.

Research Aim

To evaluate effectiveness, efficiency, safety, and patient experience of case management approaches to the care of people who call the emergency ambulance service frequently; and gain an understanding of barriers and facilitators to implementation.

Research Objectives

1. Develop an understanding of predicted mechanisms of change to underpin the evaluation
2. Describe epidemiology of people who use 999 frequently
3. Evaluate case management approaches to the care of people who call the 999-ambulance service frequently in terms of:
    - further emergency contacts (emergency ambulance calls, Emergency Department attendance, emergency admissions to hospital)
    - effects on other health and social care services
    - adverse events (deaths; injuries; serious medical emergencies; police arrests)
    - costs of intervention and subsequent use of health and social care

For peer review only - http://bmjopen.bmj.com/site/about/guidelines.xhtml

- patient experience of intervention and subsequent use of health and social care

4. Identify challenges and opportunities associated with using case management models, including features associated with success, and develop theories about how case management works in this population.

METHODS AND ANALYSIS

Study design

STRategies to manage Emergency ambulance Telephone Callers with sustained High needs – an Evaluation using linked Data (STRETCHED) is a mixed methods 'natural experiment' evaluation. We will use anonymised linked routine outcomes and qualitative data in four UK ambulance services with one intervention and one control site in each service.

Work package 1: Logic model

We will develop a logic model in consultation with stakeholders during a face-to-face workshop to underpin the evaluation and inform data collection and finalisation of outcome selection. The logic model will include definition of key components of case management in this setting; predicted mechanisms of change; and possible outcomes, positive or otherwise. Stakeholders to be invited to this workshop include clinical and managerial partners working within the ambulance service, Emergency Departments, primary care, and high intensity user services from across the UK.

Figure 1 shows a draft logic model, developed from the research literature and specialist knowledge of research team members, to be used as a basis for discussion and development during WP1.

Work package 2*:* Evaluation

We will undertake an evaluation using a natural experiment cohort design in four ambulance services, using quantitative anonymised linked routine data to describe epidemiology and assess effects of the intervention on processes, outcomes, safety and costs of intervention and subsequent health and social care up to six months later, with adjustment for covariates including prior service use. We will also collect qualitative data from focus groups and interviews in each intervention site about the views and experiences of stakeholders (commissioners, emergency and non-acute health and social care providers) regarding acceptability, successes, and challenges of case management approaches for this group of patients, and in-depth interviews with a range of service users within the target population

For peer review only - http://bmjopen.bmj.com/site/about/guidelines.xhtml

Work package 3: Synthesis of quantitative and qualitative findings

We will formally synthesise quantitative and qualitative findings from WP 2, informed by the logic model developed in WP1 and in consultation with stakeholders included in the research team

For peer review only

For peer review only - http://bmjopen.bmj.com/site/about/guidelines.xhtml

Setting

The study will be undertaken in four ambulance services, identified through a survey of practice across the UK, where both case management and traditional 'within-service' models are in place in different areas within the service. Emergency ambulance services in the UK provide emergency are to '999' callers which is free at the point of use. Following mergers in the 1990s, services cover large areas, each serving a population of between 3 and 9 million people; most cover urban and rural locations and provide a range of emergency responses including telephone advice, attendance of an emergency vehicle for face-to-face assessment by a paramedic or Emergency Medical Technician, and conveyance to hospital for further care when judged clinically appropriate.

Characteristics of control and intervention site care in the four ambulance service sites included in STRETCHED are summarised in Table 1.

Table 1: Sites within participating services and key features of control (usual) and intervention (case management) site care

	Control site: Usual care	Intervention site: New model of care
Care models: generic description	Aim: to reduce or stop calls made by people making high use of the 999 service<br><br>Within-service management<br>• Letter sent to GP and patient<br>• Callers flagged; care management plan developed for use in ambulance call centre to triage patient when call comes in<br>• Contact may be made with other services to intervene<br>• Police action may be requested	Aim: to address and meet complex patient needs, thereby reducing emergency contacts by people making high use of the 999<br><br>Multi-Disciplinary Team cross-sector case management<br>Usual (within-service) care plus<br>• Dedicated frequent caller nurse/prehospital care practitioner funded by CCG/HB<br>• Multidisciplinary team meetings attended by ambulance service; partnership approach with other agencies including district nursing, social workers, police, out of hours providers, mental health professionals, ED, voluntary sector, NHS 111, GPs, and Occupational Therapy to develop and share joint care plans to address patient need

For peer review only - http://bmjopen.bmj.com/site/about/guidelines.xhtml

Outcomes

1. Further emergency contacts:
    - Emergency ambulance calls
    - Emergency Department attendance
    - Emergency admissions to hospital
    - Declassification/reclassification as 'frequent caller'
2. Effects on other health and social care services
3. Adverse events, as available
    - Deaths
    - Injuries
    - Serious medical emergencies
    - Police arrests
4. Costs of intervention and subsequent use of health and social care
5. Patient experience of intervention

Data collection

We will use a parallel cohort 'natural experiment' study design to determine effects on processes and outcomes of care, using anonymised linked data from NHS Digital and the NHS Wales Informatics Service (NWIS). The variation in exposure and outcomes will allow us to carry out analysis to link effects to intervention i.e., causes [1]. We will include callers meeting the criteria for classification as 'frequent caller' by the ambulance service during 2018. We expect to recruit 300 high users per service (n=1200), allowing us to detect a change in rate of further emergency events/death of +/- 20% with 90% power and 95% confidence (see figure 2). We will finalise data items following completion of the logic model but expect to include (up to six months): further calls to emergency ambulance service, ED attendances, emergency admissions, and deaths; declassification or reclassification as 'frequent caller'; costs; and details of demographics, case mix and patterns of calls, e.g., 'out-of-hours' (evenings/nights/weekends/holidays). Historical data about prior service use will allow us to adjust analyses for differences between cohorts, strengthening this study design.

We will collect qualitative data at the intervention site within each participating ambulance service to explore perceptions of how the intervention works, what creates its effect (if any), why it might function differently in different settings and for different groups of people, and any challenges to implementation and delivery of the intervention. Subject to any restrictions associated with COVID-19, we will conduct four focus groups (supplemented where necessary by telephone or online interviews) with stakeholders – including front line staff (paramedics, call handlers), partner health and social care providers, commissioners, and managers (n=36). Focus group topic guides will cover case management delivery processes, barriers, and facilitators to changed working, perceived impact for patients, issues around diversity and terminology, strengths and weaknesses of the approach and wider organisational

For peer review only - http://bmjopen.bmj.com/site/about/guidelines.xhtml

impact across health economies, such as information sharing, communication, and continuity of care.

To ensure we gain a strong picture of patient experience and circumstances we will conduct largely unstructured interviews with people in intervention areas of each service who are referred for case management (n=32). Research paramedics based at each site will undertake interviews as far as possible. We will use this method to provide an opportunity for patients to provide their own narrative about their circumstances, experiences and views regarding their needs, service use and care received before and after the case management intervention, and the intervention itself, including terminology used. We will work with participating services to identify and invite callers to this key patient-focused element of the study. We will sample purposively to include a wide range of views and experiences, and select individuals with differing demographic characteristics, length of time they have required care and management approach provided for them to include typical and atypical patient stories. We will try to include patients from Black, Asian, and ethnic minority groups.

With respondents' consent, we will audio-record and transcribe all individual and group interviews.

Data analysis

Quantitative analysis

Ambulance services will provide NHS Digital and NHS Wales Informatics Service (NWIS) with information on recruited patients, to allow matching to anonymised data from multiple sources. We will create a single integrated study database for analysis within the Secure Anonymised Information Linkage (SAIL) Gateway (19).

We will summarise patient recruitment via a CONSORT flowchart [20], and will provide descriptive data summaries for patients, by service and by site, for demographics, call patterns and volume. Numbers of events will be dichotomised where contextually useful (for instance, to declassify or reclassify callers), and converted to rates when appropriate.

Our primary analyses will be by treatment allocated, so that patients meeting the criteria for 'frequent caller' status in an intervention (case management) site will be included in analysis in that arm of the study whether they were offered or received any intervention. The outcome measure time point will be six months from a patient's first appearance on a monthly update to the 'frequent callers' list. This will apply to patients at control sites as well as intervention sites. This time point is appropriate to allow us to detect any effect, as case management is a targeted, time limited intervention designed to work within six months.

We will use multilevel generalised mixed linear models to obtain adjusted comparison of outcomes in patients at intervention sites versus those in patients receiving usual care at control sites. Using linked routine data will allow us to gather retrospective data on service use for callers included in each cohort, strengthening comparisons

For peer review only - http://bmjopen.bmj.com/site/about/guidelines.xhtml

by enabling adjustment for historical and contemporaneous differences in service use, case mix, and demographics. Reflecting an initial focus on comparing (any) case management approach with the service list approach, coding for sites will initially assume that outcomes under usual care are similar across ambulance services, as are outcomes at intervention sites. However, we will use background information on the service interventions, the descriptive summaries and formal testing, based on flexible and nested site coding, to test and modify this assumption, as appropriate – for instance, allowing intervention outcomes to vary systematically from service to service. The precise form of models will reflect the nature of the variable under consideration (logistic models for binary variables; negative binomial models for count variables; linear models for raw and transformed measurement outcomes, including rates); the multilevel element of the model will reflect the geographical clustering of sites within services. The adjusted comparisons will incorporate information on covariates and factors based on demographic and case-mix data. We will formalise these analyses in a Statistical Analysis Plan, using the relevant Swansea Trials Unit Standard Operating Procedure, which will detail conventions on model fitting (including inclusion and exclusion rules for covariates and factors), management of missing data, and the reporting of outcomes.

Health Economic evaluation

Using a cost-benefit framework, we will compare the implementation cost of case management approaches to the potential benefits of "avoiding expenditure" in subsequent health and social care resource utilisation from a public sector perspective. The overall resource implications for case management approaches for frequent callers will include intervention implementation costs, costs of 999 calls and the costs arising from utilisation of other health and care services in the 6-month follow-up period.

The costs of case management interventions will be derived through discussion with relevant staff and observation of operational processes and procedures, considering staff time and other resources required to deliver the intervention Each resource element will be costed using appropriate unit costs derived from published sources, and/or consultation with relevant finance staff.

The costs associated with a sample of the 999 calls in each study area will be determined by identifying staff inputs, along with materials, equipment, therapies, and other relevant resources used during the response to the call and the processes involved in its management and completion. A cost profile for 999 calls will be developed for each geographical area and summarised as cost per 999 calls.

The utilisation of other health and care services by the study cohorts in the control and intervention sites will be captured by routine data sources (i.e., NHS Digital and the SAIL databank), and costed using appropriate published unit costs. The inclusion of mental health and social care resources will be subject to routine data availability and quality validation.

For peer review only - http://bmjopen.bmj.com/site/about/guidelines.xhtml

To assess the rate of return on investment, we will describe changes in service utilisation over time, using changes in volume of calls and their occurrence. Our cost-benefit analysis will include net present value and internal rate of return estimates to assess the relative value for money of case management approaches in managing frequent callers of ambulance 999 services and whether the cost of case management services is shared across organisations. We will undertake a series of sensitivity analyses to estimate the effect of parameter variation on baseline findings and to determine the extent to which case management of these callers is an efficient use of public funds.

Qualitative analysis:

Analysis of focus group and interview transcripts will be carried out by members of the research team with the two Public and Patient Involvement (PPI) representatives, alongside input from the research paramedics. We will remove all identifiable data from interview transcripts before analysis. We will use a data-driven thematic approach to analysis which generates themes from the implicit and explicit ideas within participants' accounts [21]. We will follow the six stages of analysis described by Braun and Clarke [22]: data familiarisation, generating initial coding, searching for themes, reviewing themes, defining, and naming themes, producing a report. During the analysis process, we will check emerging coding and themes with the wider research team and members of the patient panel. We will assess data saturation [23,24,25] during analysis to see whether new themes are emerging by reviewing the codebook (the working document that records updates in changes to codes). We will also assess whether we have an adequate range of participants reflected in our purposive sample. Analysis of the large volume of data (transcripts, field, and observation notes) generated by the interviews will be supported by use of NVivo, computer assisted qualitative data analysis software.

Synthesis

Synthesis and reporting of quantitative and qualitative findings will be informed by the STRETCHED logic model. Quantitative data will be used to draw conclusions about comparative costs and effects; qualitative data will help us to understand and interpret these results, and to generate theories about how the new models of care are working. We will bring together key themes from across all the work packages on the effectiveness, attitudes, barriers, and facilitators to case management. We will interpret overall effectiveness and cost-effectiveness results in the light of analysis from the qualitative data about which components of case management e.g., care plan, timing of interventions, shared decision-making is perceived to work well and for whom. We will use the logic model to inform the synthesis of results which will be considered and interpreted at a joint meeting of the Research Management and Patient Advisory Groups.

ETHICS

We have received approval from the Health Research Authority (19/WA/0216) and NHS R&D permissions at all participating organisations.

For peer review only - http://bmjopen.bmj.com/site/about/guidelines.xhtml

Patients will not be approached for consent to participate in the main effectiveness study (WP2) as identifying information will be held within ambulance services only and not shared with the research team. With information governance permissions in place, retrospective routine data will be linked anonymously by NHS Digital and NWIS using a split file approach, for analysis within the SAIL Gateway [26].

A small sample of current patients who are calling frequently will be invited by the participating ambulance services to give consent and participate in one-to-one interviews. We recognise that these patients may be vulnerable and will take care to ensure that interviews are carried out sensitively, in a place of the respondent's choice or by telephone/online e.g., Zoom and by appropriately trained researchers at each site.

PATIENT AND PUBLIC INVOLVEMENT

We have worked extensively with public contributors to develop a comprehensive approach to active involvement of patients and the public at all study stages [25]. We are proposing a layered approach so that people can be involved at strategic and local levels in line with their interest, experience, and health. Our aim is to enable active and meaningful involvement throughout to enhance research quality, rigour, and ethical standards, in line with the Swansea Trials Unit SOP on service user involvement [25] and best practice [27]. Our public co-applicants (PG, BE) have been actively involved in study design and shaping the proposal. They have been involved in proposal design to undertake interviews with patients, challenging biases and assumptions in the team and helping choose of proper language to describe people who phone 999. They are members of the Research Management Group and equal partners in decisions about study implementation and dissemination.

We will also convene a Patient Advisory Panel of six individuals recruited through community groups, support agencies and third sector networks. The Panel will reflect the range of people who make frequent calls to 999 including older people, those with chronic illness and people from lower socio-economic levels. We will seek to ensure that public contributors within the study match as closely as possible the diverse population of people making frequent calls. This Panel will be a less formal route for people to contribute to specific tasks such as advising on patient facing materials, sense-checking patient results and devising dissemination materials. We will hold face-to-face or virtual meetings and visit people in their homes if that is their preference, to ensure their involvement (25, 27, 28).

We will recruit a further two public members to the independent Research Advisory Group to bring patient perspectives to oversight and scrutiny decisions. We will offer honoraria for all involvement and reimburse incurred expenses.
We will provide training for all public contributors, proposing an induction at start of involvement and training in specific skills such as Good Clinical Practice, analysis, meeting skills and dissemination skills, as contributors require [29, 30].

For peer review only - http://bmjopen.bmj.com/site/about/guidelines.xhtml

For peer review only

DISCUSSION

Evaluation of interventions that have already been implemented is challenging. It is impossible to carry out randomised controlled trials in this circumstance, frequently encountered in the real world of health services research [31]. In this circumstance alternative evaluation designs need to be used, inevitably sacrificing the ability to definitively ascribe changes observed to the intervention being evaluated – as there is always the possibility of confounding by other systematic differences between sites or patients. In the STRETCHED study we have planned a quasi-experimental study in which we will compare differences between control sites (usual care) combined and intervention (case management) sites combined. Using linked anonymised outcomes, we will be able to adjust for pervious patterns of service utilisation, thus strengthening our comparisons.

It is important to provide evidence about what works and how for this patient group, to inform the development of care. We aim, through this study, to gain robust evidence about costs, effects, safety, and stakeholder views, including patients so that the best care can be provided to improve processes and outcomes for people who make high use of the emergency ambulance service, as well as to reduce pressure on services that provide emergency care.

Competing Interests: There are no competing interests for any authors

Authors' contributions: The study concept and design was conceived by HS, AW, RWA, AP, AJ, AK, AE, BME, PG, BAE, TF, RF, AR, RP, RC, AT, BS, HH, CJP, and JS. The work will be conducted and reported by TF, RF, AR, RP, RC, HS, AP, AW, RWA, AJ, AK, AE, BME, PG, BAE, AT, BS, HH, CJP, and JS. All authors provided edits and critiqued the manuscript for intellectual content.

Funding statement: This project is funded by the National Institute for Health Research (NIHR) Health Services & Delivery Research (HS&DR) Programme (reference 18/03/02). The views expressed are those of the author(s) and not necessarily those of the NIHR or the Department of Health and Social Care

For peer review only - http://bmjopen.bmj.com/site/about/guidelines.xhtml

REFERENCES

1. The Health and Social Care Information Centre (HSCIC). Ambulance services, England 2014-15. Available from: https://digital.nhs.uk/data-and-information/publications/statistical/ambulance-services/ambulance-services-england-2014-15 (accessed 10 Aug 2020)
2. Emily Andrew, Ziad Nehme, Peter Cameron & Karen Smith (2020) Drivers of Increasing Emergency Ambulance Demand, Prehospital Emergency Care, 24:3, 385
3. Burt CW, McCaig LF, Valverde RH. Analysis of ambulance transports and diversions among US emergency departments. Ann Emerg Med 2006; 47: 317–326.
4. Association of ambulance chief executives. Annual Report 2014-15. Available from: http://aace.org.uk/wp-content/uploads/2015/10/AACE-ANNUAL-REPORT-2014-2015-FINAL-W.pdf (accessed 10 Aug 2020).
5. Keogh B and Urgent and Emergency Care Review Team. Transforming urgent and emergency care services in England Urgent and Emergency Care Review End of Phase 1 Report NHS England. Leeds; 2013. Available from: https://www.nhs.uk/NHSEngland/keogh-review/Documents/UECR.Ph1Report.FV.pdf. (Accessed 10 Aug 2020)
6. Ambulance Quality Indicators; NHS England. November 2016. Available from: https://www.england.nhs.uk/statistics/statistical-work-areas/ambulance-quality-indicators/ (accessed 10 Aug 2020)
7. Edwards M, Bassett G, Sinden L et al. Frequent callers to the ambulance service: patient profiling and impact of case management on patient utilisation of the ambulance service. Emergency Medicine Journal 2014; 32:392-396.
8. Smith D. Frequent caller: identification and management policy. Northwest ambulance service NHS trust; 2015. Available from: https://www.nwas.nhs.uk/media/387174/frequent_callers_policy_final_v2_0_ds.pdf. (Accessed 10 Aug 2020).
9. Scott, J., Strickland, A. P., Warner, K., & Dawson, P. (2014). Frequent callers to and users of emergency medical systems: a systematic review. Emergency medicine journal, 31(8), 684-691.
10. The Health and Social Care Information Centre (HSCIC). Ambulance services, England 2014-15. Available from: https://digital.nhs.uk/data-and-information/publications/statistical/ambulance-services/ambulance-services-england-2014-15 (accessed 10 Aug 2020)
11. Moe J, Kirkland S, Ospina M et al. Mortality, admission rates and outpatient use among frequent users of emergency departments: a systematic review. Emergency Medicine Journal 2015; 33:230-236.
12. Althaus F, Paroz S, Hugli O et al. Effectiveness of Interventions Targeting Frequent Users of Emergency Departments: A Systematic Review. Annals of Emergency Medicine 2011; 58:41-52. e42.
13. Gravelle H, Dusheiko M, Sheaff R et al. Impact of case management (Evercare) on frail elderly patients: controlled before and after analysis of quantitative outcome data. BMJ 2006; 334:31.

For peer review only

For peer review only - http://bmjopen.bmj.com/site/about/guidelines.xhtml

14. Agarwal, G., Lee, J., McLeod, B., Mahmuda, S., Howard, M., Cockrell, K., & Angeles, R. (2019). Social factors in frequent callers: a description of isolation, poverty, and quality of life in those calling emergency medical services frequently. BMC public health, 19(1), 684.
15. Mahmuda, S., Wade-Vallance, A., Stosic, A., Guenter, D., Howard, M., Agarwal, G., ... & Angeles, R. (2020). Understanding Why Frequent Users of EMS Call 9-1-1: A Grounded Theory Study. Health Promotion Practice, 21(3), 440-447.
16. Scott J, Strickland A, Warner K et al. Describing and predicting frequent callers to an ambulance service: analysis of 1 year call data. Emergency Medicine Journal 2013; 31:408-414.
17. Moe J, Kirkland S, Ospina M et al. Mortality, admission rates and outpatient use among frequent users of emergency departments: a systematic review. Emergency Medicine Journal 2015; 33:230-236.
18. Wassmer R, Winward L, Derlet R. Does Counselling Reduce Frequent Emergency Department Use? 2008. Available from: https://papers.ssrn.com/abstract=1093870 (accessed 10 Aug 2020).
19. Jones, K. H., Ford, D. V., Thompson, S. and Lyons, R. (2020) "A Profile of the SAIL Databank on the UK Secure Research Platform", International Journal of Population Data Science, 4(2).
20. Schulz K, Altman D, Moher D. CONSORT 2010 Statement: updated guidelines for reporting parallel group randomised trials. BMC Medicine 2010;8.
21. Guest G, MacQueen K, Namey E. Applied thematic analysis. Los Angeles Sage Publications 2012.
22. Braun V, Clarke V. Using thematic analysis in psychology. Qualitative Research in Psychology 2006; 3:77-101.
23. Marshall C, Rossman G. Designing qualitative research. Los Angeles:Sage 2016.
24. Ziebland S, McPherson A. Making sense of qualitative data analysis: an introduction with illustrations from DIPEx (personal experiences of health and illness). Medical Education 2006; 40:405-414.
25. Miles MB, Huberman AM. Qualitative Data Analysis. An Expanded Sourcebook. Second edition. Thousand Oaks, CA: Sage 1994
26. Lyons R, Jones K, John G et al. The SAIL databank: linking multiple health and social care datasets. BMC Medical Informatics and Decision Making 2009;9. doi:10.1186/1472-6947-9-3
27. Staniszewska S, Brett J, Simera I, Seers K, Mockford C, Goodland S et al. GRIPP2 reporting checklists : tools to improve reporting of Patient and Public nvolvement in Research BMJ 2017 ; 358 : j3453
28. Evans B, Bedson E, Bell P et al. Involving service users in trials: developing a standard operating procedure. Trials 2013; 14:219.
29. Crowe, S., Adebajo, A., Esmael, H. et al. 'All hands-on deck', working together to develop UK standards for public involvement in research. Res Involv Engagem 6, 53 (2020).
30. INVOLVE – What is public involvement in research? http://www.invo.org.uk/find-out-more/what-is-public-involvement-in-research-2/. (Accessed 10 Aug 2020)
31. Deaton, A., & Cartwright, N. (2018). Understanding and misunderstanding randomized controlled trials. Social science & medicine (1982), 210, 2–21.

For peer review only - http://bmjopen.bmj.com/site/about/guidelines.xhtml

Figure 1 Emergent logic model

Figure 2 ‘Natural Experiment’ Flow Diagram

For peer review only

For peer review only - http://bmjopen.bmj.com/site/about/guidelines.xhtml

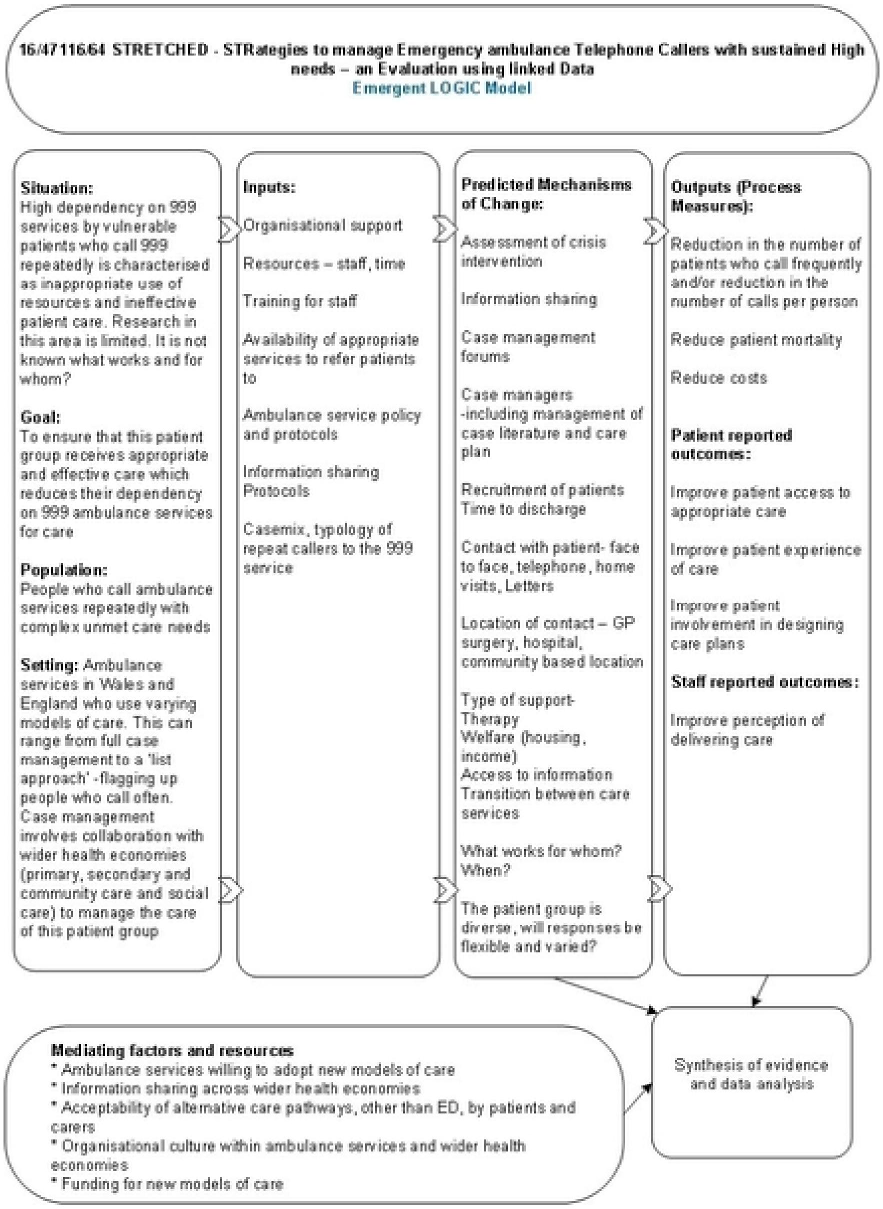

Figure 1

37x51mm (300 x 300 DPI)

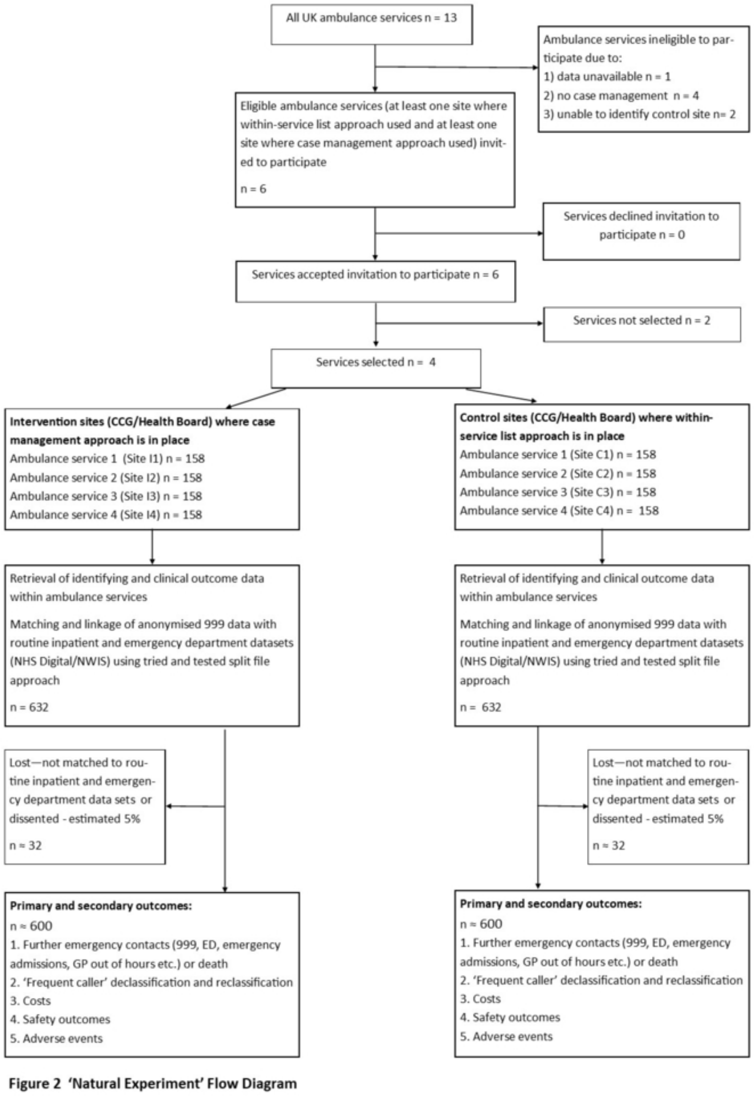

Figure 2

62x89mm (300 x 300 DPI)

CONSORT 2010 checklist of information to include when reporting a randomised trial*

Section/Topic	Item No	Checklist item	Reported on page No
**Title and abstract**			
	1a	Identification as a randomised trial in the title	1
	1b	Structured summary of trial design, methods, results, and conclusions (for specific guidance see CONSORT for abstracts)	2
**Introduction**			
Background and objectives	2a	Scientific background and explanation of rationale	3
	2b	Specific objectives or hypotheses	4
**Methods**			
Trial design	3a	Description of trial design (such as parallel, factorial) including allocation ratio	3
	3b	Important changes to methods after trial commencement (such as eligibility criteria), with reasons	N/A
Participants	4a	Eligibility criteria for participants	7
	4b	Settings and locations where the data were collected	7,8
Interventions	5	The interventions for each group with sufficient details to allow replication, including how and when they were actually administered	7
Outcomes	6a	Completely defined pre-specified primary and secondary outcome measures, including how and when they were assessed	8
	6b	Any changes to trial outcomes after the trial commenced, with reasons	N/A
Sample size	7a	How sample size was determined	9
	7b	When applicable, explanation of any interim analyses and stopping guidelines	9, 10
Randomisation:			N/A
Sequence generation	8a	Method used to generate the random allocation sequence	7
	8b	Type of randomisation; details of any restriction (such as blocking and block size)	7
Allocation concealment mechanism	9	Mechanism used to implement the random allocation sequence (such as sequentially numbered containers), describing any steps taken to conceal the sequence until interventions were assigned	N/A
Implementation	10	Who generated the random allocation sequence, who enrolled participants, and who assigned participants to interventions	N/A
Blinding	11a	If done, who was blinded after assignment to interventions (for example, participants, care providers, those	

For peer review only

		assessing outcomes) and how	
	11b	If relevant, description of the similarity of interventions	7
Statistical methods	12a	Statistical methods used to compare groups for primary and secondary outcomes	9,10
	12b	Methods for additional analyses, such as subgroup analyses and adjusted analyses	10
**Results**			
Participant flow (a diagram is strongly recommended)	13a	For each group, the numbers of participants who were randomly assigned, received intended treatment, and were analysed for the primary outcome	9,10
	13b	For each group, losses and exclusions after randomisation, together with reasons	Attached figure 2
Recruitment	14a	Dates defining the periods of recruitment and follow-up	9
	14b	Why the trial ended or was stopped	N/A
Baseline data	15	A table showing baseline demographic and clinical characteristics for each group	N/A
Numbers analysed	16	For each group, number of participants (denominator) included in each analysis and whether the analysis was by original assigned groups	N/A
Outcomes and estimation	17a	For each primary and secondary outcome, results for each group, and the estimated effect size and its precision (such as 95% confidence interval)	10
	17b	For binary outcomes, presentation of both absolute and relative effect sizes is recommended	N/A
Ancillary analyses	18	Results of any other analyses performed, including subgroup analyses and adjusted analyses, distinguishing pre-specified from exploratory	N/A
Harms	19	All important harms or unintended effects in each group (for specific guidance see CONSORT for harms)	N/A
**Discussion**			
Limitations	20	Trial limitations, addressing sources of potential bias, imprecision, and, if relevant, multiplicity of analyses	9,10
Generalisability	21	Generalisability (external validity, applicability) of the trial findings	9,10
Interpretation	22	Interpretation consistent with results, balancing benefits and harms, and considering other relevant evidence	9,10
**Other information**			
Registration	23	Registration number and name of trial registry	N/A
Protocol	24	Where the full trial protocol can be accessed, if available	N/A
Funding	25	Sources of funding and other support (such as supply of drugs), role of funders	16

*We strongly recommend reading this statement in conjunction with the CONSORT 2010 Explanation and Elaboration for important clarifications on all the items. If relevant, we also recommend reading CONSORT extensions for cluster randomised trials, non-inferiority and equivalence trials, non-pharmacological treatments, herbal interventions, and pragmatic trials. Additional extensions are forthcoming: for those and for up to date references relevant to this checklist, see www.consort-statement.org.

For peer review only - http://bmjopen.bmj.com/site/about/guidelines.xhtml

For peer review only
